# Supplementary material for: The Shifting Demographic Landscape of Pandemic Influenza
Source: PLoS One. 2010 Feb 26;5(2):e9360. doi: 10.1371/journal.pone.0009360 (PMC2829076; doi:10.1371/journal.pone.0009360)
Supplement: Text S1 — Supporting methods and analysis. (0.42 MB PDF) [file pone.0009360.s001.pdf]

The Shifting Demographic Landscape of Pandemic Influenza: Supporting Information

Shweta Bansal, Babak Pourbohloul, Nathaniel Hupert, Bryan Grenfell, Lauren Ancel Meyers

1 Supplementary Methods

1.1 Derivation of Residual Degree Distribution

The full derivation for the degree distribution of the residual network model proceeds as follows:

As the extended residual network is defined by the uninfected nodes of the previous epidemic, a proportion α of the infected nodes of the previous epidemic, and all the edges joining them, we define the degree distribution for the extended residual network as:

$$p_{res}\left(k_r\right) = \frac{p_{res}^{uninfected}\left(k_r\right) + \alpha p_{res}^{infected}\left(k_r\right)}{\sum_{k_o} p_{k_o}\eta_{k_o} + \alpha \sum_{k_o} p_{k_o}\left(1-\eta_{k_o}\right)}. \tag{1}$$

We find that the $p_{res}^{uninfected}\left(k_r\right)$ can be found by

$$p_{res}^{uninfected}\left(k_r\right) = \sum_{k_o \geq k_r} p_{k_o}\eta_{k_o} p_{res}\left(k_r|k_o\right) \tag{2}$$

where $p_{res}\left(k_r|k_o\right)$ is the probability that a node in the extended residual network will have degree k_r given that it had a degree of k in the original network. This condition distribution can Dbe calculated as

d

$$p_{res}(k_r|k_o) = \left(\begin{array}{c} k_o \\ k_r \end{array}\right)(u_1 + (1-u_1)\alpha)^{k_r}\left[(1-u_1)(1-\alpha)\right]^{k_o-k_r} \tag{3}$$

as discussed in the main text. Following Bayes rule, $p_{res}^{uninfected}\left(k_r\right)$ is then the sum of the product of the probabilities that a node in the extended residual network has degree k_r given that it had original degree k_o , $(p_{res}(k_r|k_o))$, the probability that the node of original degree k_o was uninfected in the first epidemic, (η_{k_o}), and the probability that the node had original degree k_o , (p_{k_o}) . In a similar way, $p_{res}^{infected}\left(k_r\right)$ can be calculated as:

$$p_{res}^{infected}\left(k_r\right) = \sum_{k_o \geq k_r} p_{k_o}\left(1-\eta_{k_o}\right) p_{res}\left(k_r | k_o\right) \tag{4}$$

Thus, by substituting equations 2 and 4 into equation 1, we have:

$$p_{res}\left(k_r\right) = \frac{\sum\limits_{k_o \geq k_r} p_{k_o}\eta_{k_o} p_{res}\left(k_r|k_o\right) + \alpha \sum\limits_{k_o \geq k_r} p_{k_o}\left(1-\eta_{k_o}\right) p_{res}\left(k_r|k_o\right)}{\sum\limits_{k_o} p_{k_o}\eta_{k_o} + \alpha \sum\limits_{k_o} p_{k_o}\left(1-\eta_{k_o}\right)} \tag{5}$$

And lastly, by substituting equations 3 into equation 5, we have:

$$p_{res}\left(k_r\right) = \frac{\sum\limits_{k_o \geq k_r} p_{k_o}\eta_{k_o} \binom{k_o}{k_r} (u_1+(1-u_1)\alpha)^{k_r} [(1-u_1)(1-\alpha)]^{k_o-k_r} + \alpha \sum\limits_{k_o \geq k_r} p_{k_o}\left(1-\eta_{k_o}\right) \binom{k_o}{k_r} (u_1+(1-u_1)\alpha)^{k_r} [(1-u_1)(1-\alpha)]^{k_o-k_r}}{\sum\limits_{k} p_k \eta_k + \sum \alpha p_k (1-\eta_k)}.$$

2 Supplementary Analysis

2.1 Incidence Data from Influenza Pandemics

Here, we list the confidence intervals for the odds ratio of attack rates in school-age children to adults. We note that the definitions of the school-age children and adult age groups vary across the studies and sometimes slightly differ from our own.

Year	Strain	Odds Ratio of School-Age Children to Adults [CI]	Location	Source	Data Details
1918	A (H1N1)	1.71 [0.89, 3.29]	10 U.S. cities	Frost et al, 1929	Data collected by the Publich Health Service; influenza case counts per 100 individuals in canvassed group (which included at least 5% of each city's population) by age
1918	A (H1N1)	1.71 [0.89, 3.29]	Baltimore	Collins et al, 1957	cases counts per 100 individuals in canvassed group by age; illnesses counted included influenza, grippe, pneumonia and colds in bed
1918	A (H1N1)	1.91 [1.02, 3.57]	U.S. cities	Collins et al, 1931	cases counts per 100 individuals in canvassed group by age; illnesses counted included influenza, grippe, pneumonia and colds in bed
1957	A (H2N2)	2.35 [1.31, 4.22]	Louisiana	Gilbert et al, 1962	study reports summarized age-specific influenza attack rates by age from 5 high school family survey study
1957	A (H2N2)	2.57 [1.38, 4.76]	U.K.	Gani et al , 2005	age-specific attack rate by age
1957	A (H2N2)	2.45 [1.35, 4.47]	Missouri	Davis et al, 1970	study reports age-specific attack rates of ILI in families of students who attended a high school in Kansas City, MO
1968	A (H3N2)	1.0 [0.57, 1.76]	Missouri	Davis et al, 1970	study reports age-specific attack rates of ILI in families of students who attended a high school in Kansas City, MO
2009	A (H1N1)	1.75	Mexico	Secretaria de Salud	reported case counts; we normalized case counts by age group size in Mexico
2009	A (H1N1)	5.06	USA	CDC	reported case counts as of May 19, 2009; we normalized the case counts by age group size in the US
2009	A (H1N1)	15.63	Japan	Nishiura et al, 2009	reported confirmed case counts as of June 1, 2009; we normalized case counts by age group size in Japan.
1919	A (H1N1)	0.68 [0.25, 1.86]	Baltimore	Collins et al, 1957	cases counts per 100 individuals in canvassed group by age; illnesses counted included influenza, grippe, pneumonia and colds in bed
1959	A (H2N2)	0.9 [0.5, 1.64]	Louisiana	Gilbert et al, 1962	study reports summarized age-specific influenza attack rates by age from 5 high school family survey study
1959	A (H2N1)	0.71 [0.39, 1.31]	Ohio	CDC Report, 1962	based on questionnaire survey of OH Dept. of Health personnel and their families in Jan 1960; reported percent ill "which includes those personals with a serious cold"
1969	A (H3N2)	0.14 [0.02, 0.89]	unknown	Taylor, 1971	reported rate per 100 at risk by age for influenza or ILI; based on interview by general practioner; we note that children in this study were 5-14 year olds, and adults were 15-64

Tab. 1: Details on attack rate data used for Figure 3

2.2 Data on Immunity to Influenza

Here we review empirical data from literature on effects of immunity due to previous influenza infection.

Sample Size	Subtype/Strain	Year of study	Reinfection Rate	Timescale	Source
	A H1N1	1920	8%	1 year	Jordan, 1927
94	A H3N2	1972	9%	1 year	Noble et al, 1974
43	A H3N2	1973	16%	1 year	Foy et al, 1976
100,000	various	1984-2002	7.4%	1 year	Finkenstadt et al, 2005
138	A H3N2	1977	5%	1 year	Pease, 1987
~600-30,000	A H1N1	1918	6-51%	$<$ 1 year	Barry et al., 2008

Tab. 2: Empirical data on effects of immunity due to previous influenza infection

2.3 Invasion Threshold with Immunity

When invading a population which has previously been (partially) infected by a similar strain, a pathogen faces an epidemic threshold for the a subsequent outbreak, which is a critical value of transmissibility above which a second epidemic is possible in the the same population. This critical transmissibility threshold is a function of the transmissibility of the first season pathogen, T_1, and the loss of immunity, α, and can be calculated as:

$$Tc_2 = \frac{\langle k_r \rangle}{\langle k_r\left(k_r - 1\right)\rangle}$$

where, $\langle k_r \rangle$is defined by $\sum_k kp_{res}\left(k\right)$ and $p_{res}(k)$ is the residual network degree distribution for our urban network model. $T_2 = Tc_2$ is equivalent to $R_e = 1$. In a naive population ($\alpha = 1$), the transmissibility of an invading pathogen can be quite low for successful invasion. As you consider scenarios with increasing immunity (i.e. as α decreases), the epidemic threshold for an invading pathogen is increasingly higher. In particular, Figure 1, shows the decreasing value of Tc_2 with increasing α for a first-season epidemic of $R_0 = 1.1, 1.6, 2.1$.

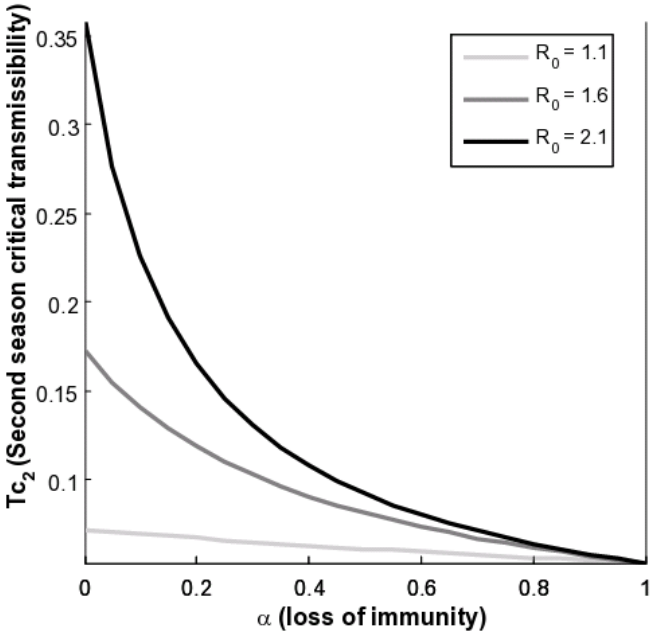

Fig. 1: We calculate the pathogen transmissibility threshold required for a second season invasion in our urban network model with loss of partial immunity α and a first season epidemic with reproductive number $R_0 = 1.1, 1.6, 2.1$.

2.4 Sensitivity to Contact Patterns

The contact patterns of the populations in the pandemics of the last century (1918, 1957 and 1968) may have differed from those of today. Here, we explore sensitivity to changes in population structure of the shift that occurs in the risk of infection from an intial pandemic season to a subsequent season. We consider two probability distributions of contacts (degree distributions) different than that of our urban network model: a) the Mossong et al study [4] is an empirical survey which reports daily conversational and physical contacts of individuals in European countries, and b) the Eubank et al study [1] is a synthetic population simulated based on data from the city of Portland, Oregon that reports daily contacts. In addition to differences in contact structure, these studies incorporate differences in the demographic structure of the population. Specifically, the Eubank et al study is made up of 20% children and 51% adults, while the Mossong et al study is made up of 28% children and 48% adults,

compared with 23% children and 61% adults in our population model.

Figure 2 below shows that both populations still exhibit the shift in infection risk from high contact individuals to moderate contact individuals. Thus given higher contact rates for school-age children than adults, a shift in age-specific incidence can be expected from the prior age group to the latter.

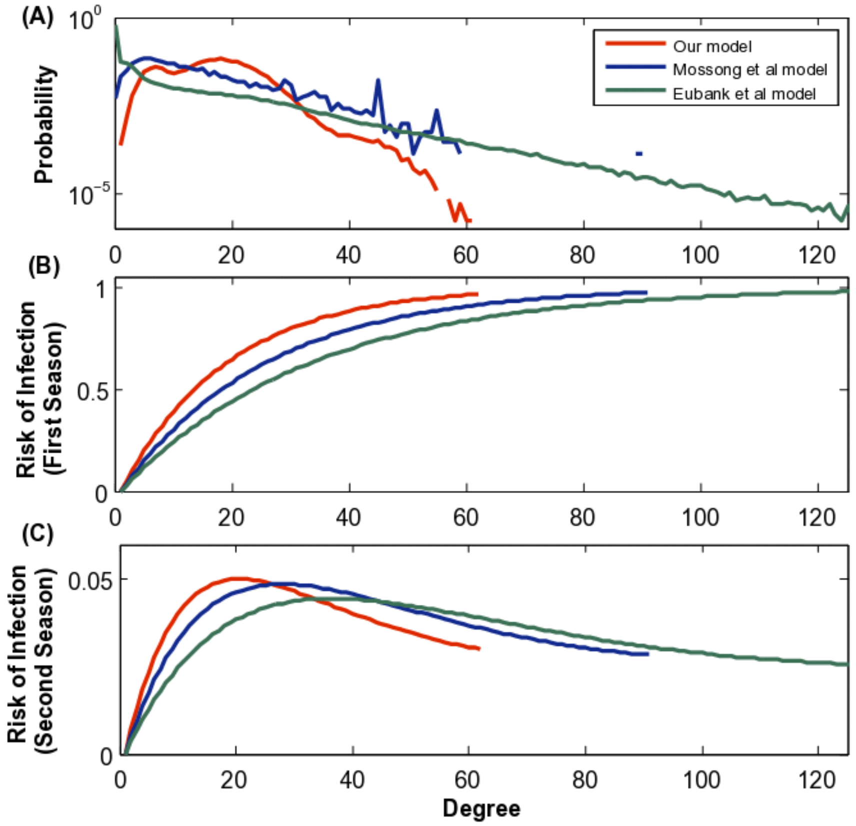

Fig. 2: We calculate risk of infection in first and second season epidemics for populations with varying contact patterns. (A) Degree distributions from our urban network model (based on [3], the Mossong et al study [4], and the Eubank et al study [1]. (B) The degree-based risk of infection for a naive population (no prior immunity) for each network for $R_0 = 1.6$ (C) The degree-based risk of infection for a subsequent season (of $R_e = 1.05$), following a first season epidemic (of $R_0 = 1.6$) with partial immunity ($\alpha = 0.05$) for each network. The shift in risk of infection from high contact individuals to moderate contact individuals in a partially immune population is still evident.

2.5 Sensitivity to the Reproductive Number

The reproductive number for the H1N1/09 pandemic has been estimated to be between 1.22 and 2.3 [2, 6, 5]. Figure 3 shows that our results on the shift in risk of infection are robust to varying R_0 values.

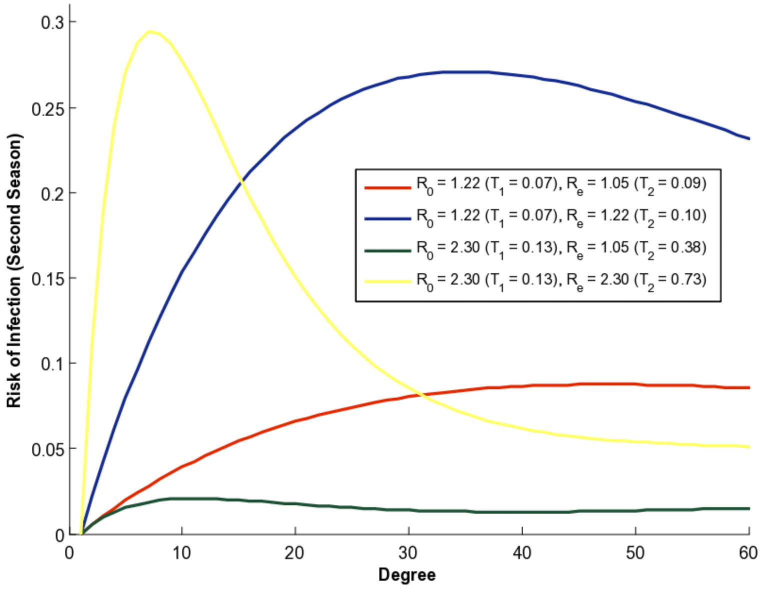

Fig. 3: Risk of Infection for a range of initial reproductive number (R_0) and effective reproductive number (R_e) for a second season. The shift in risk of infection is still evident.

2.6 Sensitivity to Demographic Changes

Here, we consider disease consequences in a population with partial immunity from a previous year as well as demographic changes. We ignore births and deaths as they occur at either end of the population age scale and do not impact contact patterns to a significant degree. Instead, we focus on aging in the population and the impact of this on population contact patterns. Specifically, we allow aging of individuals at the boundaries of age (and thus contact) classes: 4 year olds age to 5 year olds (and move from the low-contact class of toddlers to the high-contact class of school-aged children), 18 year olds age to be 19 years old (and move from the high-contact class of school-aged children to the moderate-contact class of adults), and adults of age 64 age to 65 years of age (and move from the moderate-contact class of adults to the low-contact class of elderly.) In Figure 4, we see the differences in the degree distribution of the contact networks with and without these demographic changes in populations

with full, partial and no immunity ($\alpha = 0, 0.5, 1$). As the left panel shows, aging only impacts the degree distribution in a small way. To quantify the disease impact of these changes, we calculate the size of the epidemic in the populations with and without aging for various levels of immunity and also find that the impact is not dramatic.

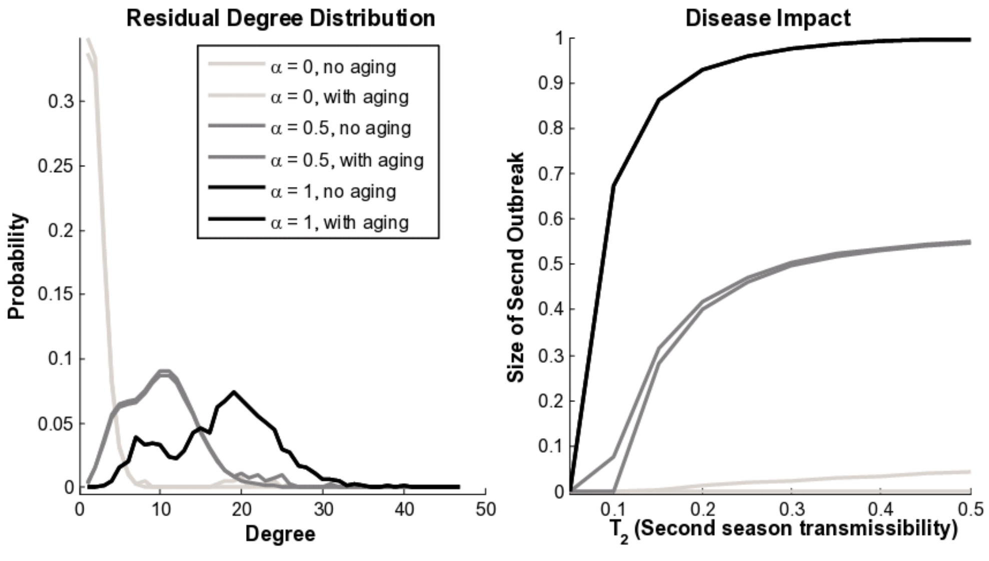

Fig. 4: Impact of the demographic process of aging on the contact network structure with varying values of immunity (left panel) and on the disease consequences in the population (right panel.)

2.7 Comparing Stochastic Simulations to Percolation Model

Finally, we compare the predictions from our percolation-based model to stochastic simulations. The standard percolation theory framework is based solely on the degree distribution of the population contact network. Comparison to stochastic simulations allow us to quantify the impact of higher order structural properties (such as clustering in edges, degree correlations, etc.) Below, we compare the predictions from our percolation model to that of stochastic simulations for the risk of infection in the second season by degree and find congruent results.

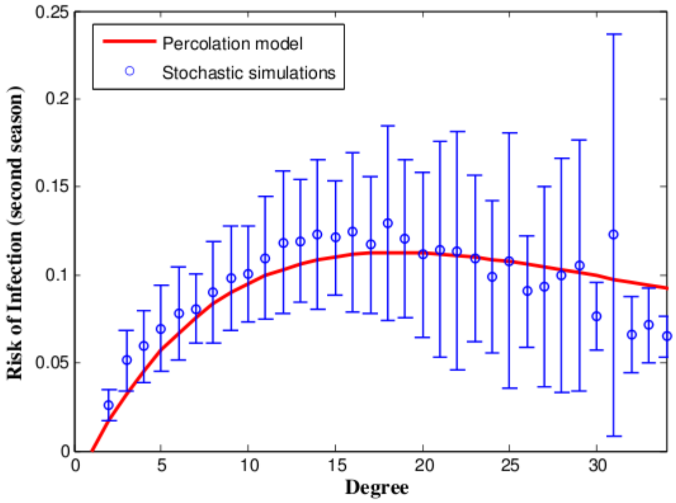

Fig. 5: Comparison of stochastic simulations and analytical percolation model predictions for risk of infection in second season based on indivdiual degree.

References

[1] S. Eubank. Synthetic data products for societal infrastructures and proto-populations: Data set 2.0. Technical Report NDSSL-TR-07-003, Network Dynamics and Simulation Science Laboratory, Virginia Polytechnic Institute and State University, 2008.

[2] Christophe Fraser, Christl A. Donnelly, Simon Cauchemez, William P. Hanage, Maria D. Van Kerkhove, T. DÃ©irdre Hollingsworth, Jamie Griffin, Rebecca F. Baggaley, Helen E. Jenkins, Emily J. Lyons, Thibaut Jombart, Wes R. Hinsley, and Nicholas C. Grassly. Pandemic Potential of a Strain of Influenza A (H1N1): Early Findings. *Science*, 324:1557–1561, 2009.

[3] L.A. Meyers, B. Pourbohloul, M.E.J. Newman, D.M. Skowronski, and R.C Brunham. Network theory and sars: predicting outbreak diversity. *J. Theo. Biol*, 232:71–81, 2005.

[4] J. Mossong, N Hens, M Jit, P Beutels, and K. et al. Auranen. Social contacts and mixing patterns relevant to the spread of infectious diseases. *PLoS Medicine*, 5:3, 2008.

[5] H. Nishiura, C. Castillo-Chavez, M. Safan, and G. Chowell. Transmission potential of the new influenza A(H1N1) virus and its age-specificity in Japan. *Eurosurveillance*, 14:22, 2009.

[6] B. Pourbohloul, A. Ahued, B. Davoudi, R. Meza, L.A. Meyers, D.M. Skowronski, I. Villasenor, P. Kuri, F. Galvan, P. Cravioto, J. Trujillo, D.J. Earn, J. Dushoff, D. Fisman, J. Edmunds, N. Hupert, S.V. Scarpino, D.M. Patrick, and R.C. Brunham. Initial human transmission dynamics of the pandemic (H1N1) 2009 virus in North America. *Influenza and Other Respiratory Viruses*, 3 (5):215–22, 2009.
